# 7-O-sulfonation redirects luteolin’s target preference from furin to NS2B/NS3 and enhances anti-dengue activity

**DOI:** 10.3389/fmicb.2026.1931544

**Published:** 2026-10-02

**Authors:** Huini Chen, Zizhao Lao, Yuhang Huo, Liangwen Yu, Wen Zhou, Geng Li

**Affiliations:** 1 Science and Technology Innovation Center, Guangzhou University of Chinese Medicine, Guangzhou, Guangdong, China; 2 Chinese Medicine Guangdong Laboratory, Hengqin, Guangdong, China; 3 School of Medicine, The Sixth Affiliated Hospital of South China University of Technology (Nanhai District People’s Hospital of Foshan), Foshan, Guangdong, China

**Keywords:** antiviral, dengue virus, luteolin derivatives, molecular docking, NS2B/NS3 protease

## Abstract

**Background:**

Dengue virus (DENV) infection remains a global health threat with no approved small-molecule antiviral for clinical use. Luteolin, a natural flavonoid, exhibits anti-dengue activity but suffers from poor solubility and moderate potency. The 7-O position is the primary site of metabolic glucuronidation, whereas the 5-O position is sequestered by intramolecular hydrogen bonding, offering structurally distinct positions for derivatization.

**Methods:**

Five site-selective derivatives of luteolin (three 5-O- and two 7-O-substituted) were synthesized. Their anti-DENV activity was evaluated in Vero cells by qRT-PCR (measuring viral RNA abundance) and plaque assays, and cytotoxicity was determined using the CCK8 method. For mechanistic exploration, enzymatic inhibition kinetics against NS2B/NS3 and furin were measured, and target engagement was further assessed via molecular docking and cellular thermal shift assays (CETSA).

**Results:**

Among the derivatives, compound 5, bearing a 4-fluorobenzenesulfonyl group at the 7-O position, exhibited the most potent anti-DENV activity (EC_50_ = 0.46 μM) and the highest selectivity index (SI = 28.5), representing an 18-fold increase in antiviral potency over luteolin. In enzymatic assays, compound 5 showed preferential inhibition of the viral NS2B/NS3 protease (*K*_*i*_ = 11.54 μM) over the host furin (*K*_*i*_ = 93 μM). Molecular docking and thermal stabilization assays provided correlative evidence supporting target engagement with both proteases.

**Conclusion:**

Site-selective 7-O-sulfonation of luteolin yields compound 5, a derivative with improved anti-DENV activity and selectivity that shows preferential NS2B/NS3 inhibition in enzymatic assays. This study provides a structural framework for the further optimization of flavonoid-based antiviral agents.

## 1 Introduction

Dengue fever, transmitted by *Aedes aegypti* mosquitoes, is one of the most rapidly spreading mosquito-borne viral diseases worldwide. Its incidence has surged nearly 30-fold over five decades, and the virus is now endemic in more than 130 countries, placing an estimated 4 billion people at risk (Finch et al., 2025; He et al., 2026). Despite the unprecedented global dengue burden in 2024, with millions of reported cases and thousands of deaths across all WHO regions, no small-molecule antiviral has yet been approved for clinical use (EClinicalMedicine, 2024; Haider et al., 2025). The NS4B inhibitor NITD-688 is currently the only candidate in Phase 2a clinical trials, while other candidates such as AT-752 and JNJ-1802 have been discontinued (McBride et al., 2026). Although the tetravalent vaccine Qdenga has been approved in several countries and demonstrated 61.2% efficacy against symptomatic dengue and 84.1% against hospitalization over 4.5 years, its efficacy varies by serotype and baseline serostatus, wanes over time, and safety concerns including anaphylaxis have emerged in mass vaccination programs (Ranzani et al., 2026; Tricou et al., 2024). Consequently, patient management remains largely confined to symptomatic and supportive treatments.

The DENV genome is a single-stranded positive-sense RNA of approximately 11 kb, encoding three structural proteins–capsid (C), precursor membrane (prM), and envelope (E)–and seven non-structural proteins (NS1, NS2A, NS2B, NS3, NS4A, NS4B, and NS5) (Bhatnagar et al., 2021). The dengue virus life cycle begins with receptor-mediated endocytosis and pH-dependent fusion, which releases the viral RNA genome into the cytoplasm. Subsequently, the NS2B/NS3 complex serves as an enzymatic hub for replication. NS2B activates the NS3 protease to cleave the viral polyprotein at four cytosolic sites, while the NS3 helicase couples NTP hydrolysis to RNA unwinding during RNA synthesis. NS2B/NS3 also recruits NS5 and NS4B to assemble the functional replication complex on endoplasmic reticulum membranes. During the late stage, the host protease furin cleaves prM at the conserved -R-X-R/K-R- motif in the trans-Golgi network, releasing the pr peptide and triggering the fusogenic conformational change of the E protein, thereby rendering virions infectious. Together, NS2B/NS3-driven replication and furin-mediated maturation represent two critical, temporally coordinated steps essential for productive DENV infection (Khanra et al., 2025; Nanaware et al., 2021; Sinha et al., 2024; Tse et al., 2022).

Previous studies established that luteolin, a bioactive flavone isolated from *Viola yedoensis*, exhibits potent *in vitro* anti-DENV activity and confers partial protection in DENV-infected AG129 mice (Peng et al., 2017). Mechanistically, luteolin non-competitively inhibits the host furin protease, impairing prM cleavage and leading to elevated prM levels in culture supernatants. However, selection of luteolin-resistant viral variants revealed T79R and I114M substitutions in prM and NS2B, respectively, suggesting that luteolin may also directly target the viral NS2B/NS3 protease complex. These findings indicate that luteolin acts as a dual-target inhibitor of both furin and NS2B/NS3, which may underlie its antiviral efficacy (Peng et al., 2018).

In recent years, significant progress has been made in the development of NS2B-NS3 protease inhibitors, including peptides, peptidomimetics, covalent small molecules, and natural products (João et al., 2024). For example, structure-based optimization yielded pyrazole ester-based covalent inhibitors WSL-75 and WSL-84 with improved activity over the lead compound WSL-01, while an N-methylcytisine thio derivative (compound 6) was shown to inhibit both DENV E protein-mediated viral entry and NS2B-NS3 protease activity, exhibiting a dual antiviral mechanism (Cheng et al., 2022; Lin et al., 2024). Among natural products, flavonoids have garnered considerable interest as antiviral leads due to their structural diversity and low toxicity (Aguiar-Pech et al., 2025). Multiple flavonoids, including quercetin, luteolin, kaempferol derivatives, and apigenin, have been reported to inhibit NS2B-NS3 by binding to the active site or allosteric pockets. Notably, quercetin, curcumin, and myricetin have been characterized as non-competitive inhibitors that modulate enzyme activity through interactions with the NS2B cofactor or by inducing conformational changes in NS3 (Saqallah et al., 2022).

Nevertheless, luteolin has significant pharmacokinetic drawbacks, including poor aqueous solubility that leads to low *in vivo* exposure and limits its clinical potential. In the molecular modification of luteolin, the 5-O and 7-O position are preferentially chosen for derivatization owing to differences in chemical reactivity, metabolic stability, and biological activity (Lo et al., 2021; Robotham and Brodbelt, 2013). The 7-O position is more reactive and the primary site of metabolic glucuronidation. Hence, its modification represents a key strategy to improve oral bioavailability. By contrast, the 5-O position forms an intramolecular hydrogen bond with the 4-carbonyl group, which stabilizes the molecular conformation and is essential for antioxidant activity (Wu et al., 2024; Xu et al., 2022). Among various derivatization strategies, sulfation introduces a negatively charged sulfonate group that enhances aqueous solubility, increases molecular polarity, and can modify target-binding affinity through electrostatic interactions (Alshehri and Jones, 2024). Therefore, rational selection of the 5-O or 7-O position enables targeted improvements in solubility, stability, or pharmacological activity. Despite the promising anti-DENV activity of luteolin, systematic strategies that leverage site-selective modification to redirect its target preference and to improve its potency and selectivity remain under-explored.

Based on these considerations, we hypothesized that sulfonation at the 7-O position would confer greater selectivity for the viral NS2B/NS3 protease over the host furin than modification at the 5-O position. The 7-O position is more solvent-exposed and capable of forming hydrogen bonds with NS2B/NS3 residues, whereas the 5-O is constrained by intramolecular H-bonding with the 4-carbonyl. Moreover, a 7-O arylsulfonyl substituent, such as 4-fluorobenzenesulfonyl, may engage additional hydrophobic and π–π interactions within the NS2B/NS3 pocket (Kouretova et al., 2017; Mahalapbutr et al., 2025). The present study was designed to synthesize a series of 5-O- and 7-O-position luteolin derivatives and to evaluate their anti-DENV activities, cytotoxicity, and potential target engagement profiles. By addressing the objective, we aim to provide a structural basis for the rational optimization of flavonoid-based anti-DENV agents.

## 2 Materials and methods

### 2.1 Cells and virus

Vero cells (obtained from Yuanjing Biology, YC-A001) were verified by species-specific multiplex qPCR and verified negative for cross-contamination with other mammalian cell lines and tested negative for mycoplasma contamination. Vero cells were grown and maintained in Dulbecco’s modified Eagle’s medium (DMEM; Thermo Fisher Scientific, Waltham, USA) at 37 °C in 5% CO_2_. Compounds were dissolved in 100% dimethyl sulfoxide (DMSO) and diluted in culture medium before each assay, with the final DMSO concentration adjusted to ≤0.1% (v/v) in all treated cultures. DENV-2 strain NGC (GenBank accession number KM204118.1) was provided by Jian-guo Wu (Jinan University). All virus strains were grown in C6/36 cells and the supernatants were stored at −80 °C. All cell experiments involving viruses were conducted in accordance with institutional biosafety guidelines under biosafety level-2 (BSL-2) conditions. All subsequent assays were performed under consistent experimental conditions, including the same batch of Vero cells, DENV-2 NGC strain, culture medium, and compound preparation protocol. Appropriate vehicle (DMSO) and untreated controls were included in each assay as specified in the respective method sections.

### 2.2 Cytotoxicity measurement

Cells were grown at 6 × 10^3^ cells/well in 96-well culture plates and treated with compounds at concentrations ranging from 0.39 to 200 μM in twofold serial dilutions. The concentration range was selected to encompass a broad spectrum for accurate CC_50_ determination, with the upper limit based on preliminary solubility tests. The control group was treated with an equal volume of DMSO. After 48 h, 10% CCK8 reagent was added to each well, the plate was incubated for 1 h and absorbance values were determined at 450 nm. The half-maximal Cytotoxic Concentration (CC_50_) was calculated by nonlinear regression analysis of dose-response curves using GraphPad Prism 8.0. All experiments were performed with three independent biological replicates. Each biological replicate consisted of three technical replicates per concentration.

### 2.3 Anti-DENV assay

Cells were seeded in 12-well plates at a density of 2 × 10^5^ cells/well and pre-treated with various concentrations of test compounds (0.15–200 μM) for 2 h. The concentration range for antiviral assays was selected based on the cytotoxicity profiles of each compound to ensure that the tested concentrations did not exceed the CC_50_. The 2 h pre-treatment and 48 h post-infection incubation times were chosen based on preliminary time-course experiments indicating that this interval allowed optimal virus replication while maintaining cell viability. Subsequently, the cells were infected with DENV-2 at a multiplicity of infection (MOI) of 1 for 2 h at 37 °C. An MOI of 1 was selected to achieve consistent infection efficiency across experiments while avoiding excessive viral cytopathic effects. Following removal of the viral inoculum, the cells were replenished with fresh medium containing the same compound concentrations and incubated until 48 h post-infection (hpi). The intracellular viral RNA levels were then determined using qRT-PCR. The EC_50_ value, defined as the concentration that reduces viral RNA yield by 50%, was calculated from the dose-response curves. A DMSO treated infected group served as the virus only control. Untreated cells (without virus and without compound) were included as blank controls for normalization. All experiments were performed with three independent biological replicates. Each biological replicate included three technical replicates. All experimental conditions were kept consistent across all antiviral assays to ensure comparability.

### 2.4 RNA isolation and qRT-PCR

Viral RNA was isolated using a standard protocol (CWBIO, Guangzhou, China), and qRT-PCR was performed using SYBR Green (Bio-Rad, California, USA) according to the manufacturer’s protocol. Primers used for DENV-2 E gene detection were Forward primer 5′-CAGGCTATGGCACTGTCACGAT-3′, Reverse primer 5′-CCATTTGCAGCAACACCATCTC-3′. Primers used for GAPDH gene detection were Forward primer 5′-AAGGCTGTGGGCAAGG-3′, Reverse primer 5′-TGGAGGAGTGGGTGTCG-3′. Cycling conditions were 94 °C for 2 min, followed by 40 cycles of 94 °C for 15 s and 55 °C for 30 s. GAPDH was used as the housekeeping gene and relative quantification was performed using the 2^−ΔΔCT^ (cycle threshold) method. All experiments were performed with three independent biological replicates. Each biological replicate included three technical replicates.

### 2.5 Protease inhibition assay

For both NS2B/NS3 and furin protease assays, the compound concentrations, substrate concentrations, and incubation times were selected based on preliminary dose-finding experiments to ensure that enzymatic activity remained within the linear range. For NS2B/NS3 protease: Recombinant DENV-2 NS2B/NS3 protease (provided by the School of Pharmacy, China Pharmaceutical University) was used in all experiments. In a 96-well plate, 50 nM NS2B/NS3 protease was incubated with compound 5 at final concentrations of 0 (DMSO control), 1.56, and 3.12 μM in assay buffer (100 mM HEPES pH 7.0, 0.5% Triton X-100, and 0.5 mM CaCl_2_) at room temperature for 30 min. The fluorogenic substrate Benzoyl-Nle-Lys-Arg-Arg-AMC (Nanjing Peptide Biotech Ltd.,) was then added at concentrations of 20, 50, 80, 100, and 200 μM. Fluorescence intensity was measured at an excitation wavelength of 370 nm and an emission wavelength of 460 nm at 37 °C. Readings were taken every 2 min for a total duration of 30 min. Initial reaction velocities (V_0_) were determined from the linear portion of each progress curve. Kinetic parameters were obtained by fitting the data to the Michaelis-Menten equation using nonlinear regression (GraphPad Prism 8.0). The mode of inhibition was determined by Lineweaver-Burk double-reciprocal plots (1/V vs. 1/S), and the inhibition constant (*K*_*i*_) was derived from the secondary plot of slope versus inhibitor concentration. DMSO control served as the negative control.

For furin protease: Furin (NEB) was used at 1 U per reaction. In a 384-well plate, furin was incubated with compound 5 at final concentrations of 0 (DMSO control), 50, and 70 μM in reaction buffer containing 100 mM HEPES pH 7.0, 0.5% Triton X-100, and 0.5 mM CaCl_2_ at room temperature for 30 min. The fluorogenic substrate Pyr-Arg-Thr-Lys-Arg-AMC (Bachem) was then added at concentrations of 50, 100, 200 and 400 μM. Fluorescence intensity was measured at an excitation wavelength of 370 nm and an emission wavelength of 460 nm at 37 °C. Readings were taken every 5 min for a total duration of 1 h. Kinetic parameters and inhibition constants were determined using the same nonlinear regression and Lineweaver-Burk analysis methods as described above for NS2B/NS3. All experiments were performed with three independent biological replicates. Each biological replicate included three technical replicates.

### 2.6 Plaque forming assay

Vero cells were seeded overnight at 2 × 10^5^ cells/well in a 12 well plate and infected with the supernatant at 37 °C for 2 h. After incubation, the inoculum was removed and cells were immediately replenished with plaque medium (Sigma, St. Louis, USA) supplemented with 0.8% methylcellulose. Infected cells were cultured for 7 days. After incubation, plaque medium was removed, cells were fixed with 4% formaldehyde for 2 h and stained with 0.5% crystal violet for 30min. Data are presented as mean ± SD. Statistical significance was determined by one-way ANOVA followed by Dunnett’s *post-hoc* test for comparisons with the DMSO control. All plaque assays were performed with three independent biological replicates. Each biological replicate included three technical replicates.

### 2.7 Western blotting

The separated proteins were transferred to PVDF membranes (Millipore) and nonspecific binding on the membranes was blocked with 5% BSA in PBST for 1 h. Membranes were incubated with dengue virus NS3 protein antibody or furin antibody as an internal control overnight at 4 °C. Detection was performed with HRP-conjugated anti-mouse antibody for 1 h at room temperature with shaking. Densitometric quantification was performed using ImageJ. Band intensities were measured as integrated density values after background subtraction, normalized to GAPDH, and expressed relative to the DMSO-treated infected control (set as 100%). Data are presented as mean ± SD. Statistical significance was determined by one-way ANOVA with Dunnett’s *post-hoc* test versus the DMSO control. All Western blotting were performed with three independent biological replicates.

### 2.8 CETSA

Cellular thermal shift assays was performed as described above (Chen et al., 2020), with minor changes. Briefly, Vero cells were harvested, washed with PBS, and resuspended in detergent-free buffer (25 mM HEPES, pH 7.0, 20 mM MgCl_2_, 2 mM DTT) supplemented with a cocktail of protease and phosphatase inhibitors. The cell suspension was lysed by sonication and centrifuged at 20,000 × *g* for 20 min at 4 °C to separate the soluble fraction from the cell debris. In the CETSA melting curve experiment, cytolysis was diluted with detergent-free buffer and split into two aliquots prior to DMSO (equivalent volume of compound 5) or 20 μM compound 5 treatment.

The 20 μM concentration was selected as it represented a concentration approximately 43-fold above the antiviral EC_50_. After treatment at room temperature for 30 min, each sample was divided into 11 aliquots of 50 μl/tube, heated at 33.1 °C–52.1 °C for 3 min and immediately cooled at room temperature for 3 min. The heated cell extract was centrifuged at 20,000 × *g* for 20 min at 4 °C to separate the soluble fraction from the pellet. After centrifugation, the supernatant was analyzed by blotting with antibody. The highest signal intensity within the DMSO treatment group (consistently occurring at the lowest temperature) as a 100% reference for each protein target. Densitometric analysis was performed using ImageJ software. Band intensities were quantified as integrated density values after uniform background subtraction. The apparent aggregation temperature (Tagg) was calculated by non-linear regression using a sigmoidal dose-response curve fitting model in GraphPad Prism 8.0. For each biological replicate, Tagg was derived from the inflection point of the melting curve. The mean Tagg and standard deviation (SD) were calculated from three independent biological replicates. Statistical significance of the Tagg shifts between compound 5 and DMSO control was assessed using the two-tailed unpaired Student’s *t*-test for comparing fitted curves. *P*-value of <0.05 was considered to indicate a statistically significant shift. DMSO-treated samples served as vehicle controls in all experiments. All experiments were performed with three independent biological replicates.

### 2.9 Molecular docking

Molecular docking was performed using AutoDock 4.0. Compound 5 was drawn in ChemDraw, energy-minimized using the MM2 force field, and converted to PDB format. The crystal structures of NS2B/NS3 (PDB: 2FOM) and furin (PDB: 5MIM) were downloaded from the Protein Data Bank. Water molecules and bound ligands were removed using PyMOL. For each protein, AutoDockTools was used to add polar hydrogens, compute Gasteiger charges, assign AD4 atom types, and generate grid maps. For NS2B/NS3 (PDB: 2FOM), the grid box was defined with 126 × 126 × 126 points and a spacing of 0.375 Å, yielding a physical box size of 46.875 × 46.875 × 46.875 Å^3^. Centered at (*x* = 0.409, *y* = −17.041, *z* = 15.722) Å, covering the substrate-binding pocket. For furin (PDB: 5MIM), the same grid parameters were used, with the box centered at (*x* = 35.379, *y* = −37.864, *z* = −0.394) Å to encompass the catalytic domain. For compound 5, rotatable bonds were defined and the torsion root was identified, and the ligand was saved in PDBQT format. Docking calculations were performed using the Lamarckian genetic algorithm with 100 independent runs. The best pose was selected based on the lowest binding free energy score and cluster analysis in AutoDock Tools. To validate the reliability of the molecular docking protocol, the co-crystallized ligand was extracted from the binding site and re-docked into the same protein structure. The root-mean-square deviation (RMSD) between the top-scoring docking pose and the crystallographic conformation was calculated based on all heavy atoms of the ligand. An RMSD value of ≤2.0 Å was considered indicative of a successful docking protocol.

### 2.10 Statistical analysis

Statistical analyses were performed using GraphPad Prism 8.0. All data are presented as mean ± SD from three independent biological replicates (*n* = 3). Biological replicates refer to independent experiments performed on different days with separately prepared cell cultures and compound dilutions. Technical replicates refer to repeated measurements within the same experiment. Prior to statistical analysis, the assumptions of normality and homogeneity of variance were verified using the Shapiro-Wilk test and Levene’s test, respectively. If normality or homogeneity of variance was violated, data were analyzed using the Mann-Whitney U test or Welch’s ANOVA, as appropriate. Two-group comparisons were analyzed by two-tailed unpaired Student’s *t*-test, and multiple comparisons by one-way ANOVA with Dunnett’s *post-hoc* test versus the DMSO control. Statistical significance was set at *p* < 0.05. Significance levels are indicated as *p < 0.05, **p < 0.01, and ***p < 0.001, ns, not significant (*p* > 0.05). For quantitative comparisons, all bar graphs display mean ± SD from three independent biological replicates.

### 2.11 Ethics statement

No animal studies were performed in this manuscript.

## 3 Results

### 3.1 Synthesis and structural characterization of luteolin derivatives

A series of luteolin derivatives were synthesized via structural modification at the 5-O and 7-O position (Supplementary Figure 1). Specifically, three novel 5-O-substituted derivatives (compounds 1–3) and two novel 7-O-substituted derivatives (compounds 4 and 5) were prepared (Figure 1). None of the five compounds have been previously reported in SciFinder, thereby expanding the chemical space of luteolin-based analogs. The physicochemical and spectral data for each compound are presented (Table 1 and Supplementary Figure 2).

**FIGURE 1:**
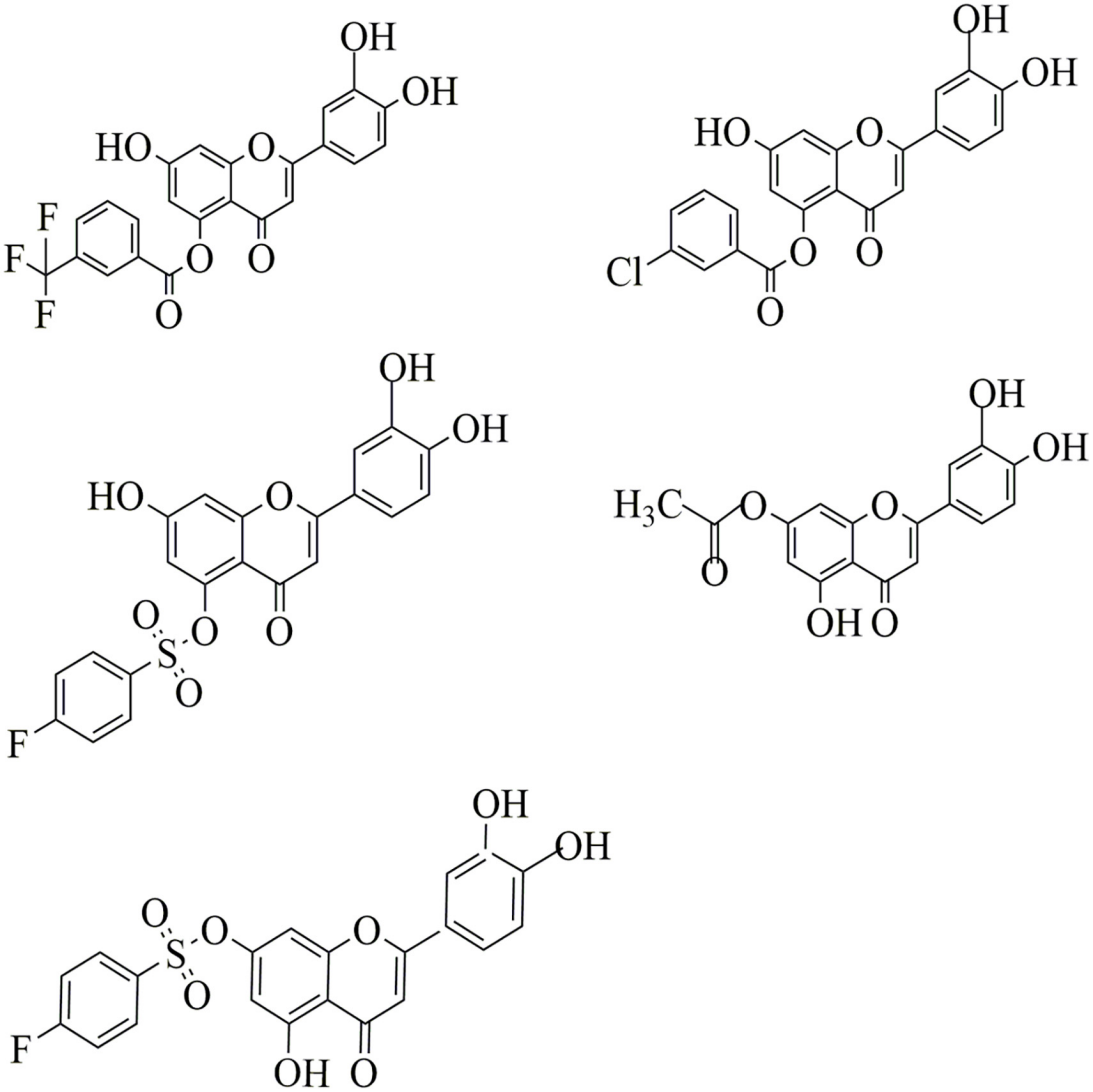
Chemical structures of luteolin derivatives (1–5).

**TABLE 1:** Properties of compounds luteolin and luteolin derivatives.

Compound	Position	Property	MW (g/mol)
Luteolin	–	Yellow, powder	286.24
Compound 1	5-O	Light yellowish-green, powder	458.06
Compound 2	5-O	Light yellow, powder	424.03
Compound 3	5-O	Yellowish-white, powder	444.03
Compound 4	7-O	Yellowish-green, powder	328.07
Compound 5	7-O	Brownish-yellow, powder	445.03

### 3.2 Cytotoxicity and antiviral activity (inferred from viral RNA abundance) of luteolin and luteolin derivatives

The cytotoxic effects of luteolin, luteoloside, and compounds 1–5 were evaluated in Vero cells. As shown in Figures 2A–G, the half-maximal cytotoxic concentrations (CC_50_) were 85.5 ± 1.34, >200, 48.78 ± 1.10, 31.36 ± 1.05, >200, 19.65 ± 1.02, and 13.09 ± 1.10 μM, respectively. Compared with luteolin, luteoloside and compound 3 exhibited reduced cytotoxicity (CC_50_ > 200 μM), whereas compounds 1, 2, 4, and 5 showed enhanced cytotoxicity, with CC_50_ values ranging from 13.09 to 48.78 μM. These results demonstrate that structural diversification significantly modulated the cytotoxic properties of luteolin derivatives. Antiviral activity was inferred from viral RNA abundance, as quantified by qRT-PCR. As illustrated in Figures 2H–K and Table 2, the half-maximal effective concentrations (EC_50_) for luteolin and compounds 1–5 were 8.28 ± 0.23, 105 ± 1.20, 12.28 ± 0.12, >25, 54.89 ± 1.10, >25, and 0.46 ± 0.022 μM, respectively. Notably, Compound 5 exhibited the lowest EC_50_ value among the tested compounds, with an EC_50_ of 0.46 ± 0.022 μM. Compound 5 was identified as the most active derivative, with a CC_50_ of 13.09 ± 1.10 μM, an EC_50_ of 0.46 ± 0.022 μM, and a selectivity index (SI = CC_50_/EC_50_) of 28.5-substantially higher than that of the parent compound luteolin (SI = 9.1). Given its substantially improved selectivity index, compound 5 was selected for further mechanistic investigation.

**FIGURE 2:**
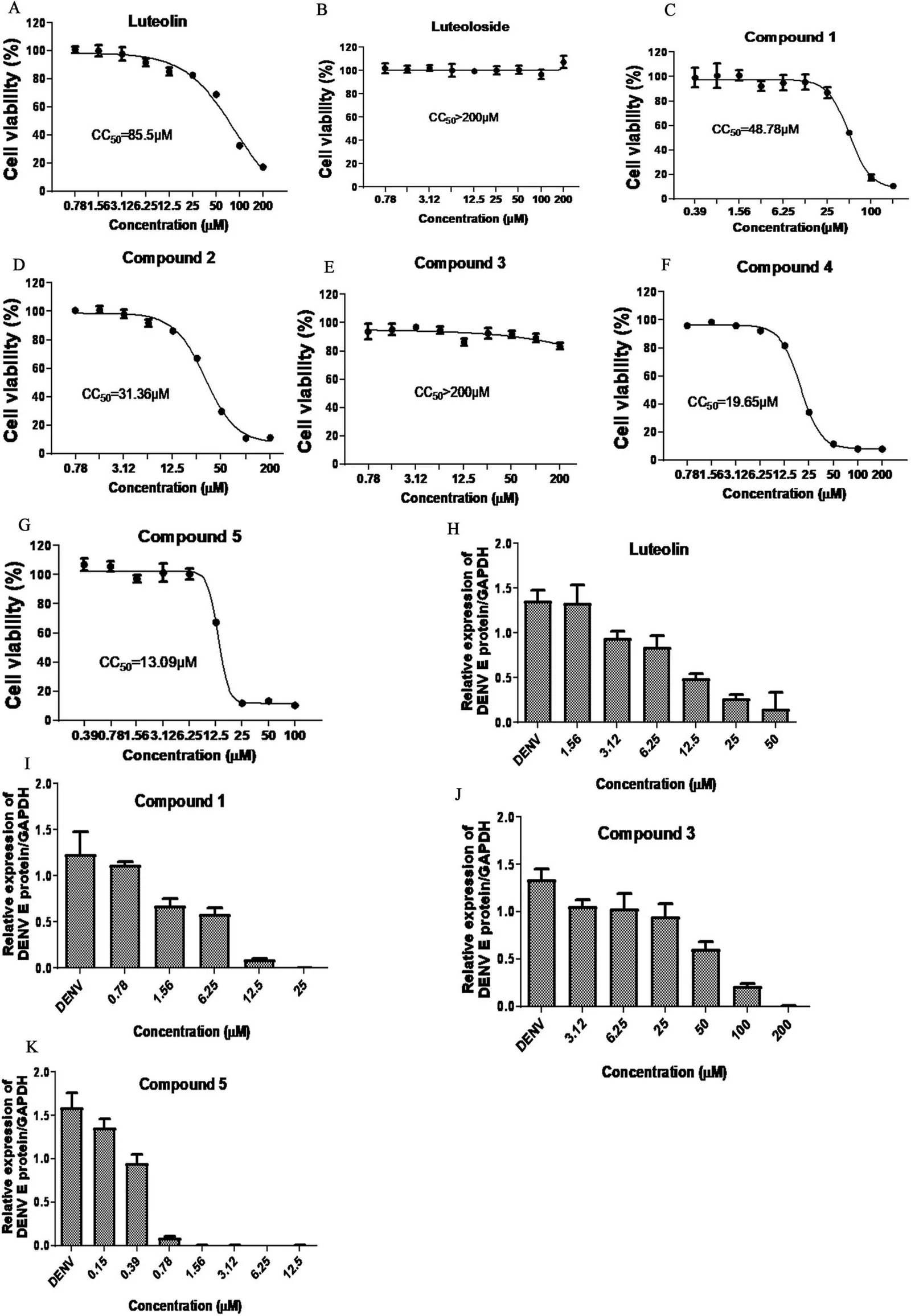
Cytotoxicity and anti-DENV activity of luteolin and its derivatives (1–5) in Vero cells. **(A–G)** Concentration-response curves for cytotoxicity. Vero cells were treated with increasing concentrations of luteolin, luteoloside, or compounds 1–5 for 48 h, and cell viability was measured using the CCK8 assay. **(H–K)** Inhibition of DENV2 replication by luteolin and its derivatives, as inferred from viral RNA abundance. Vero cells were infected with DENV-2 and then treated with the indicated compounds. Viral RNA levels were quantified by qRT-PCR at 48 h post-infection, normalized to GAPDH, and expressed relative to the DMSO-treated infected control. EC_50_ values are summarized in Table 2. All data are presented as mean ± SD from three independent biological replicates. Each biological replicate comprising three technical replicates. EC_50_ and CC_50_ values were calculated by nonlinear regression (log[inhibitor] vs. response, variable slope (four parameters)) using GraphPad Prism 8.0.

**TABLE 2:** Cell cytotoxicity and antiviral activity (inferred from viral RNA levels) of luteolin and luteolin derivatives.

Compound	EC_50_ (μM)	CC_50_ (μM)	SI
Luteolin	8.28 ± 0.23	85.5 ± 1.34	9.1
Luteoloside	105 ± 1.20	>200	>1.9
Compound 1	12.28 ± 0.12	48.78 ± 1.10	4.0
Compound 2	>25	31.36 ± 1.05	ND
Compound 3	54.89 ± 1.10	>200	>3.6
Compound 4	>25	19.65 ± 1.02	ND
Compound 5	0.46 ± 0.022	13.09 ± 1.06	28.5

Data are presented as mean ± SD from three independent biological replicates (*n* = 3). ND: not determined due to insufficient inhibition at the highest tested concentration.

### 3.3 Antiviral activity of compound 5 against DENV and other RNA viruses

Based on its superior selectivity index (SI = 28.5), compound 5 was selected for further mechanistic evaluation of its anti-DENV activity. To characterize its inhibitory effect on viral replication, western blot analysis was performed. As shown in Figures 3A,B, compound 5 suppressed the expression of DENV NS3 and prM proteins in a dose-dependent manner. Compound 5 completely inhibited the formation of viral proteins at concentrations of 0.78–12.5 μM, with approximately 50% inhibition at 0.39 μM (*p* < 0.001). The antiviral effect was quantified by plaque reduction assays. At a concentration of 0.2 μM (*p* < 0.001), the inhibition rate reached about 60%, with complete inhibition achieved at 0.39 μM (*p* < 0.001) (Figures 3C,D). Plaque reduction assays showed that the EC_50_ values of compound 5 against Zika virus, poliovirus, and influenza virus were 0.15 ± 0.011, 0.54 ± 0.035, and 4.51 ± 0.18 μM, respectively (Figure 3E). Collectively, these results demonstrate that compound 5 exerts dose-dependent antiviral activity at micromolar concentrations against DENV and the other tested viruses.

**FIGURE 3:**
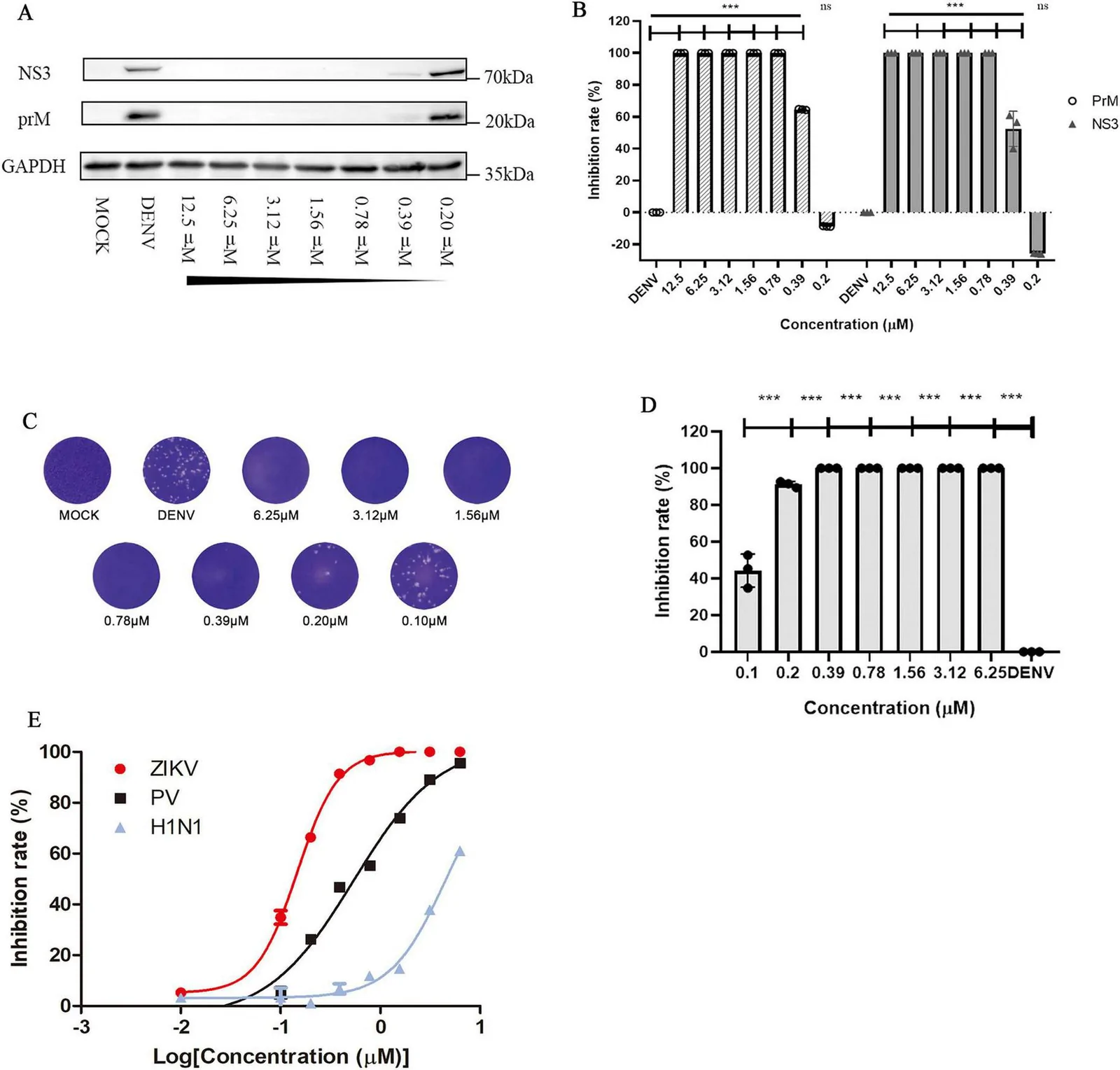
Antiviral activity of compound 5 against DENV and Other RNA Viruses. **(A)** Western blot analysis of DENV NS3 and prM protein expression in Vero cells. Cells were infected with DENV and treated with the indicated concentrations of compound 5; GAPDH was used as a loading control. **(B)** Band intensities were normalized to GAPDH and expressed relative to the DMSO-treated infected control. Data are presented as mean ± SD. Statistical significance was determined by one-way ANOVA with Dunnett’s *post-hoc* test versus the DMSO control. ***p < 0.001, ns, not significant (*p* > 0.05). **(C)** Inhibition of DENV plaque formation by compound 5 as determined by plaque assay. Partial inhibition was observed at 0.20 μM and complete inhibition at 0.39 μM. **(D)** Quantification of plaque reduction, expressed as percentage inhibition relative to the DMSO-treated infected control. Data are presented as mean ± SD. Statistical significance was determined by one-way ANOVA with Dunnett’s *post-hoc* test. ***p < 0.001. **(E)** Antiviral activity of compound 5 against Zika virus, poliovirus, and influenza virus. EC_50_ are 0.15 ± 0.011, 0.54 ± 0.035, and 4.51 ± 0.18 μM, respectively. All data are presented as from three independent biological replicates. Statistical significance was determined by one-way ANOVA with Dunnett’s *post-hoc* test. ***p < 0.001, ns, not significant (*p* > 0.05).

### 3.4 Compound 5 acted as a non-competitive inhibitor of NS2B/NS3 and furin, with higher affinity for NS2B/NS3

Kinetic analysis using Lineweaver-Burk plots revealed that compound 5 acts as a non-competitive inhibitor of both NS2B/NS3 and furin proteases, as indicated by the intersection of the lines on the *x*-axis (Figure 4). From secondary plots, the inhibition constant (*K*_*i*_) values were determined to be 11.54 μM for NS2B/NS3 and 93 μM for furin. These results indicate that compound 5 inhibits NS2B/NS3 with higher apparent affinity than furin in biochemical assays. Notably, the inhibitory activity of compound 5 against furin (*K_*i*_* = 93 μM) was weaker than that of luteolin (*K*_*i*_ = 58.6 μM). The non-competitive inhibition pattern observed in the Lineweaver-Burk plots indicates that compound 5 does not compete with the substrate for the active site. This pattern is consistent with the hypothesis that compound 5 binds to regions outside the active sites of both proteases, potentially inducing conformational changes that reduce catalytic activity.

**FIGURE 4:**
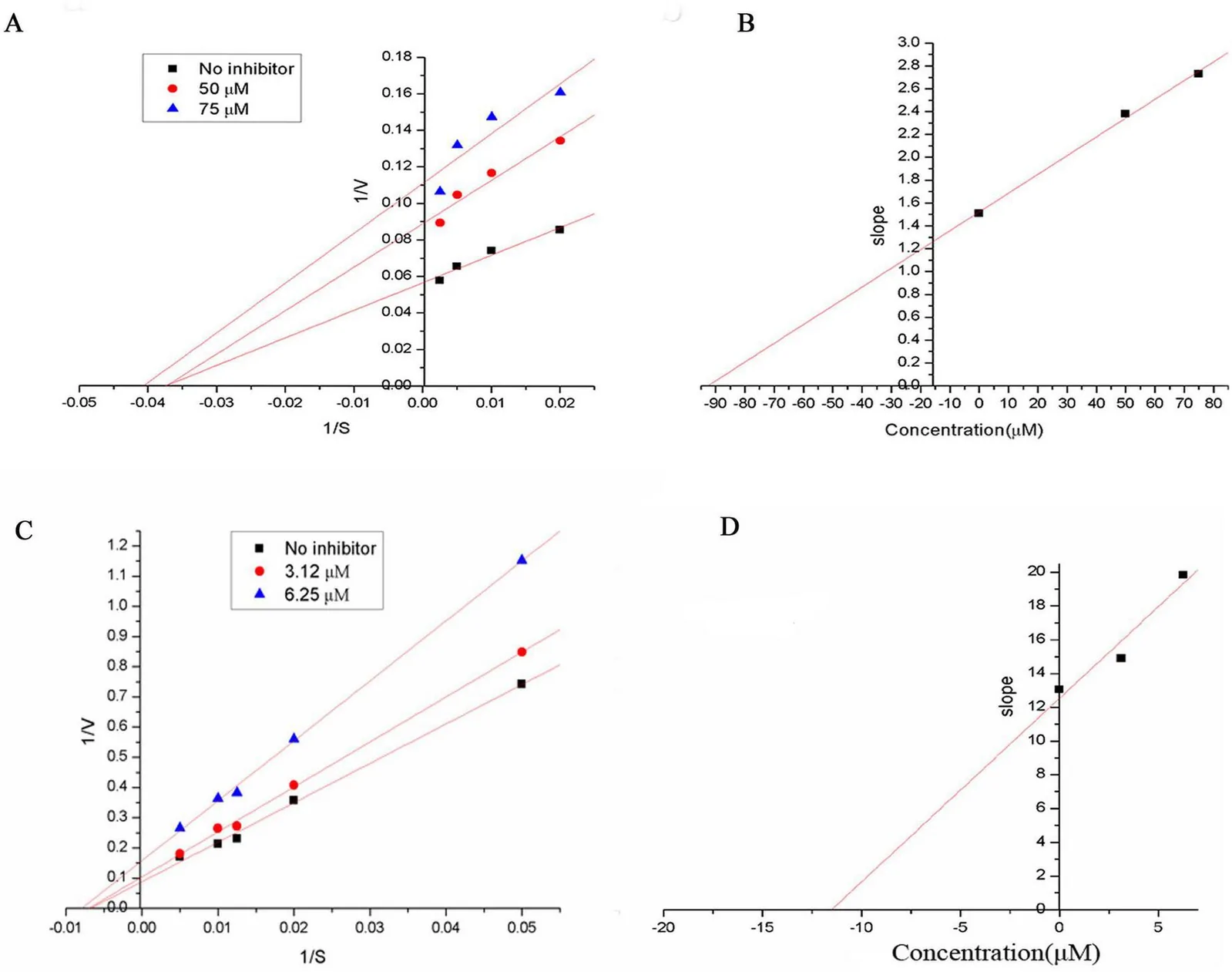
Kinetic analysis of compound 5 inhibition of furin and NS2B/NS3 proteases. Lineweaver-Burk plots are shown as double-reciprocal plots of 1/velocity versus 1/substrate. **(A)** Inhibition of furin by compound 5. **(B)** Secondary plot of slope (Vmax/Km) versus inhibitor concentration for furin. **(C)** Inhibition of NS2B/NS3 by compound 5. **(D)** Secondary plot of slope (Vmax/Km) versus inhibitor concentration for NS2B/NS3. All data are presented as mean ± SD from three independent biological replicates.

### 3.5 Molecular docking and CETSA suggest target engagement of compound 5 with NS2B/NS3 and furin

To investigate the interaction of compound 5 with its targets, molecular docking simulations and CETSA were performed. The predicted binding model for furin (Figures 5A–C) showed that compound 5 binds favorably within a hydrophobic cavity, with a binding energy of −8.72 kcal/mol (compared with −8.51 kcal/mol for luteolin; data not shown). In the optimal conformation, compound 5 formed hydrogen bonds with Asn310, Gly307, Gly265, Arg490, and Tyr571, along with multiple hydrophobic contacts. This docking-derived binding mode raises a hypothesis that the binding event may perturb the adjacent active pocket, notably affecting the active site residue Ser368, which could contribute to the reduced enzymatic activity.

**FIGURE 5:**
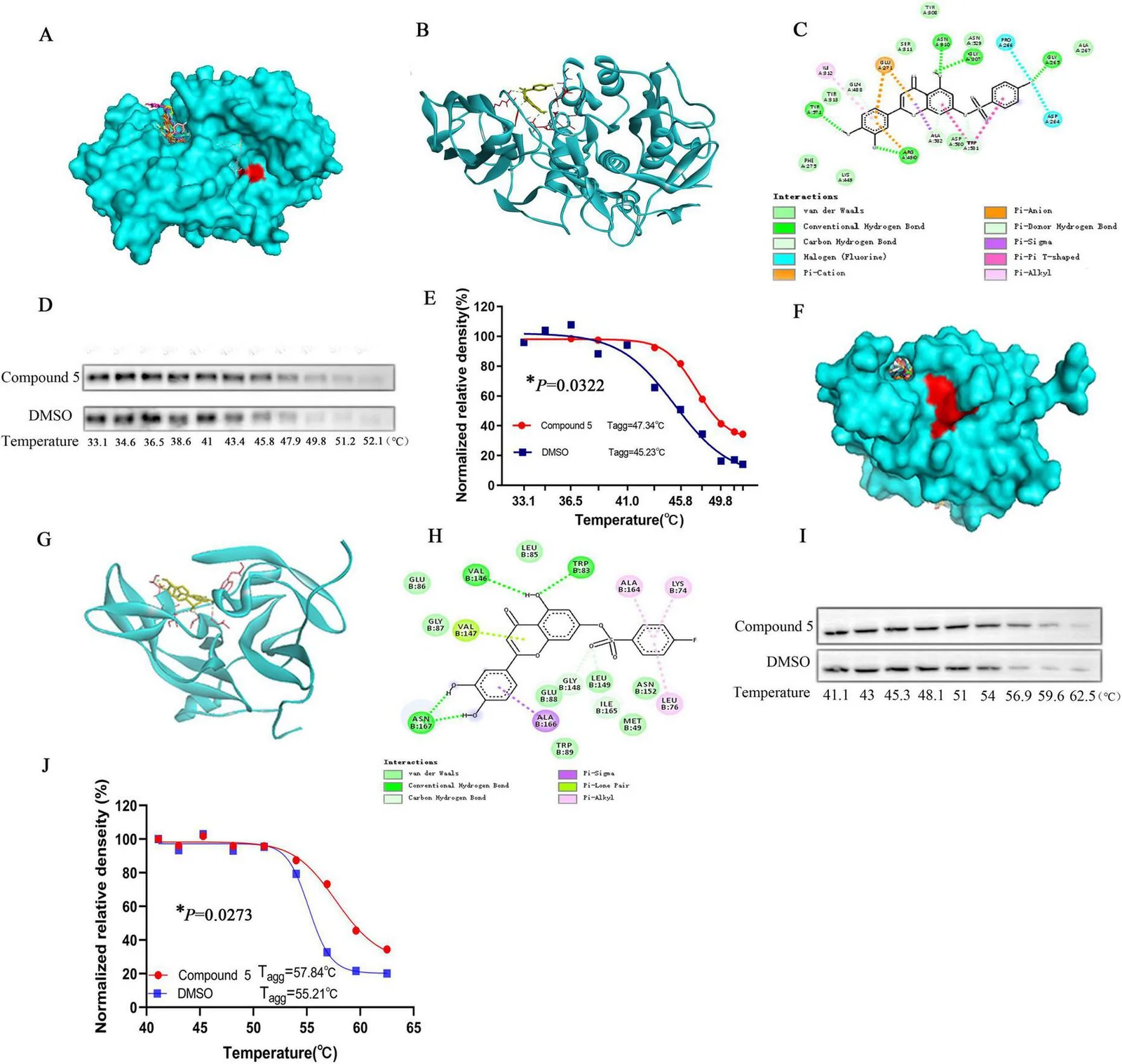
Compound 5 interacts with furin and NS2B/NS3 proteins as shown by molecular docking and CETSA. Molecular docking (**A–C** for furin; **F–H** for NS2B/NS3). **(A,F)** Surface representation of docking poses. **(B,G)** Cartoon representation of the optimal binding conformation. **(C,H)** Two-dimensional interaction diagrams showing hydrogen bonds and hydrophobic contacts. CETSA binding assays (**D**,**E** for furin; **I**,**J** for NS2B/NS3). **(D,I)** Western blot analysis of thermal stabilization in Vero cell lysates treated with DMSO or 20 μM compound 5 at the indicated temperatures. **(E,J)** Quantification of band intensities. All data are presented as mean ± SD from three independent biological replicates. Statistical significance was determined by two-tailed unpaired Student’s *t*-test for two-group comparisons. *p < 0.05.

For NS2B/NS3, docking predicted a binding energy of −10.86 kcal/mol (luteolin −7.6 kcal/mol; data not shown), suggesting a more favorable computationally estimated interaction (Figures 5F–H). In the optimal pose, compound 5 engages in hydrogen bonds with Val146, Trp83, and Asn167, as well as hydrophobic interactions. This predicted binding mode may influence the conformational changes in the nearby catalytic triad (His51, Asp75, Ser135), offering a potential structural hypothesis that warrants further experimental validation for the observed loss of protease activity. To validate target engagement in a cellular context, Vero cell lysates were treated with compound 5 or DMSO and subjected to a temperature gradient. Compound 5 increased the thermal stability (Tagg) of furin from 45.23 °C (DMSO control) to 47.34 °C (Figures 5D, E) and that of NS2B/NS3 from 55.21 °C to 57.81 °C (Figures 5I, J). These results are consistent with target engagement of compound 5 with both furin and NS2B/NS3, as evidenced by thermal stabilization in cellular lysates. Together with the biochemical inhibition data, these findings provide correlative evidence that compound 5 engages both proteases in a cellular lysate context.

## 4 Discussion

A notable observation is that, in biochemical assays, compound 5 exhibited a lower *K*_*i*_ value for NS2B/NS3 than for furin, whereas luteolin had been previously reported to show the opposite profile. This suggestive shift in relative potency warrants further direct comparative studies to confirm whether 7-O-sulfonation truly redirects target preference.

Compound 5 exhibited higher cytotoxicity (CC_50_ = 13.09 μM) than luteolin (CC_50_ = 85.5 μM). Nevertheless, it retained a favorable selectivity index, as the 18-fold improvement in antiviral activity outweighed the approximately 6.5-fold increase in cytotoxicity. Crucially, this favorable SI indicates that the gain in antiviral activity substantially outweighs the increase in cytotoxicity. If the antiviral effect were predominantly driven by nonspecific cellular toxicity, a proportional increase in both EC_50_ and CC_50_ would be expected, resulting in a similar or worse SI. Thus, the improved activity of compound 5 reflects enhanced target engagement rather than merely increased toxicity. Despite this favorable SI, the absolute cytotoxicity in normal cells remains a concern that warrants further investigation. The underlying causes are likely multifactorial. First, it may arise from off-target effects associated with the overall lipophilicity of the molecule or specific substituents. Second, although the fluorobenzenesulfonyl moiety is generally regarded as a metabolically stable pharmacophore, it may, in certain scaffold contexts, undergo cytochrome P450-mediated ipso-substitution to generate phenolic intermediates, which could be further oxidized to electrophilic quinone imine-type reactive metabolites. Such species have the potential to deplete intracellular glutathione and covalently modify proteins, thereby contributing to cytotoxic outcomes (Ma et al., 2021; Ohe et al., 1994). Future optimization efforts should prioritize substantial reduction of absolute cytotoxicity, which will be essential for further development of this compound.

The data suggest a preferential inhibition of NS2B/NS3 over furin, as supported by complementary biochemical and computational assays. The biochemical inhibition data demonstrate that compound 5 inhibits both proteases *in vitro*, with a lower *K*_*i*_ value for NS2B/NS3. CETSA provided evidence of target engagement in lysates, with compound 5 inducing a numerically larger thermal stabilization of NS2B/NS3 (ΔTagg = 2.6 ± 0.12°C) than furin (ΔTagg = 2.11 ± 0.16°C). This differential stabilization was further reflected in computational predictions, where compound 5 exhibited a markedly superior computationally estimated binding affinity for NS2B/NS3 (−10.86 kcal/mol) compared to furin (−8.72 kcal/mol). Notably, while the parent luteolin showed comparable affinity for furin (−8.51 kcal/mol), its interaction with NS2B/NS3 was substantially weaker (−7.6 kcal/mol), suggesting that the 7-O-sulfonate may confer a critical selectivity advantage, potentially through enhanced hydrophobic and hydrogen-bonding networks within the NS2B/NS3 active site. Although compound 5 retains moderate furin inhibition (*Ki* = 93 μM), this concentration is approximately 200-fold higher than its anti-DENV EC_50_, rendering furin-related off-target effects negligible at therapeutic doses. It should be noted that the present assay did not include a well-characterized positive control inhibitor for direct benchmarking; this represents a limitation that will be addressed in future studies. Collectively, these complementary lines of evidence provide a structural framework for the rational optimization of the 7-O-sulfonate scaffold toward enhanced antiviral efficacy and selectivity.

A noteworthy observation is the ∼25-fold discrepancy between the biochemical inhibition constant (*K*_*i*_ = 11.54 μM) and the cellular antiviral potency (EC_50_ = 0.46 μM). While this does not exclude NS2B/NS3 as a primary target, it merits consideration. One possible explanation is intracellular accumulation. Flavonoids are known to concentrate within cells and organelles, often reaching local levels well above extracellular concentrations. Compound 5 may similarly enrich at the viral replication complex, effectively offsetting its moderate *K*_*i*_ in the cellular context. Another possibility is that compound 5 may act through additional mechanisms. Luteolin has been reported to inhibit viral RNA-dependent RNA polymerase and to disrupt cytoskeletal organization, thereby interfering with virus release. Such pleiotropic effects, common among flavonoids, could synergize with NS2B/NS3 inhibition to yield the potent cellular EC_50_. Differences between the recombinant enzyme system and the native cellular environment, including the artificial protease construct and the presence of physiological modulators, may also contribute (Morimoto et al., 2022; Munafò et al., 2022; Sakai-Sugino et al., 2024). These hypotheses warrant further investigation.

SAR analysis revealed that compounds 1–3 (5-O-substituted) were less active than luteolin (EC_50_ = 105, >25, and 54.89 μM vs. 8.28 μM), as the intramolecular hydrogen bond between the 5-O position and the 4-carbonyl group is critical for maintaining the planar conformation. Substitution at this position disrupts this bond, altering conformation and reducing target affinity (Nath et al., 2025; Yang et al., 2026). In contrast, compound 5 (7-O-sulfonated) exhibited the most potent activity (EC_50_ = 0.46 μM), representing an 18-fold improvement over luteolin. This is attributable to the absence of intramolecular hydrogen bonding at the 7-O position and the negatively charged sulfonate group, which enables electrostatic interactions with positively charged residues in the NS2B/NS3 active site (Li et al., 2023). Molecular docking predicted a binding energy of −10.86 kcal/mol for compound 5, compared with −7.6 kcal/mol for luteolin, suggesting a more favorable computationally estimated binding interaction. The predicted binding mode involves hydrogen bonds with Val146, Trp83, and Asn167, which may contribute to the observed preferential NS2B/NS3 inhibition, although these predictions require experimental validation through mutagenesis or co-crystallization studies. The electron-withdrawing fluorine atom in the 4-fluorobenzenesulfonyl group may enhance electrostatic interactions, while the moderately increased lipophilicity could be expected to favor membrane permeation. Compound 4 (7-O-acetyl) was far less active than compound 5 (EC_50_ > 25 μM vs. 0.46 μM), suggesting that the sulfonate group may contributes substantially to the potent activity of compound 5. However, given that acetyl and sulfonate groups differ in multiple physicochemical properties (charge, hydrogen-bonding capacity, geometry, and polarity), additional analogues with structurally varied substituents (e.g., sulfonamide, phosphate, or carboxylate) would be required to systematically deconvolute the structural determinants of target binding (Li et al., 2023; Nath et al., 2025; Yang et al., 2025, 2026).

From a drug discovery perspective, compound 5 offers several advantages over existing approaches. While peptide- and protein-based furin inhibitors offer higher *in vitro* potency, their poor cell permeability limits antiviral efficacy (Lü et al., 2024; Raj et al., 2025). Small-molecule inhibitors like compound 5 offer more favorable physicochemical properties (e.g., lower molecular weight, reduced polar surface area) and potentially better intracellular access, representing a practical alternative for further development (Yang and Hinner, 2015). The 7-O-sulfonation strategy is synthetically accessible and amenable to further diversification, allowing exploration of alternative arylsulfonyl groups, sulfonamides, or bioisosteres to optimize potency, selectivity, and pharmacokinetic properties. Nevertheless, it is important to recognize that sulfonated flavonoids often exhibit poor oral bioavailability and rapid clearance due to their hydrophilic nature and susceptibility to metabolic conjugation (Li et al., 2025). Thus, extensive pharmacokinetic and toxicological evaluation remains a prerequisite for any future development.

Several limitations of the present study should be acknowledged. First, the antiviral activity was evaluated using a single DENV serotype (DENV-2); activity against DENV-1, -3, and -4 remains to be assessed. Second, the mechanisms underlying the increased cytotoxicity of compound 5 and its potential off-target effects have not been investigated. Third, the present work is entirely based on *in vitro* data; no pharmacokinetic properties (e.g., aqueous solubility, metabolic stability, or oral bioavailability) or *in vivo* efficacy studies have been conducted. Future studies should prioritize comprehensive pharmacokinetic profiling and efficacy evaluation in appropriate animal models, together with mechanistic investigations into its cytotoxicity and activity against other DENV serotypes.

In summary, the data presented in this study indicate that 7-O-sulfonation of luteolin yields a derivative with an altered target preference toward NS2B/NS3 in biochemical assays, which parallels enhanced *in vitro* anti-DENV potency and selectivity. The preferential engagement of the viral protease over furin, together with the non-competitive inhibition profile, identifies compound 5 as a promising candidate warranting further structural optimization and *in vivo* evaluation.

## 5 Conclusion

Site-selective 7-O-sulfonation of luteolin yielded compound 5, a derivative that exhibits more potent inhibition of DENV NS2B/NS3 over furin in biochemical assays and exhibits improved antiviral potency and selectivity. The available data suggest an association between compound 5 activity and NS2B/NS3 inhibition, supported by complementary biochemical, computational, and cellular target-engagement studies. Together, these findings identify 7-O-sulfonation as a promising structural strategy for modulating the target preference of luteolin and provide a foundation for further optimization of this flavonoid-based scaffold.

## Data Availability

The raw data supporting the conclusions of this article will be made available by the authors, without undue reservation.
