# Supplementary material for: 7-O-sulfonation redirects luteolin’s target preference from furin to NS2B/NS3 and enhances anti-dengue activity
Source: Front Microbiol. 2026 Oct 2;17:1931544. doi: 10.3389/fmicb.2026.1931544 (PMC13672209; doi:10.3389/fmicb.2026.1931544)
Supplement: Supplementary file 1 [file Table_1.docx]

Supplementary figure 1. Synthetic routes of luteolin derivatives (1–5).

(A–E) Synthetic schemes for compounds 1–5,respectively.

Supplementary figure 2. ¹H-NMR spectra of compounds 1–5. (A) Compound 1. (B) Compound 2. (C) Compound 3. (D) Compound 4. (E) Compound 5.
